# Bafilomycin A1 and U18666A Efficiently Impair ZIKV Infection

**DOI:** 10.3390/v11060524

**Published:** 2019-06-06

**Authors:** Catarina Sabino, Michael Basic, Daniela Bender, Fabian Elgner, Kiyoshi Himmelsbach, Eberhard Hildt

**Affiliations:** 1 Paul-Ehrlich-Institut, Department of Virology, 63225 Langen, Germany; catarina.sabino@pei.de (C.S.); michael.basic@kgu.de (M.B.); daniela.bender@pei.de (D.B.); fabian.elgner@pei.de (F.E.); kiyoshi.himmelsbach@pei.de (K.H.); 2 German Center for Infection Research (DZIF), 38124 Braunschweig, Germany

**Keywords:** Zika virus, viral life cycle, endosomal–lysosomal compartment, bafilomycin A1, U18666A, antiviral effect

## Abstract

Zika virus (ZIKV) is a highly transmissive virus that belongs to the *Flaviviridae* family, which comprises several other pathogens that threaten human health. This re-emerging virus gained attention during the outbreak in Brazil in 2016, where a considerable number of microcephaly cases in newborns was associated with ZIKV infection during pregnancy. Lacking a preventive vaccine or antiviral drugs, efforts have been made to better understand the viral life cycle. In light of this, the relevance of the endosomal–lysosomal compartment for the ZIKV life cycle was investigated. A549 and SH-SY5Y cells were infected with either the African strain (associated with mild symptoms) or the French Polynesia strain (associated with neurological complications). For both strains, the V-ATPase inhibitor, bafilomycin A1, efficiently inhibited ZIKV entry and prevented the spread of the infection by interfering with viral maturation. Additionally, affecting cholesterol metabolism and transport with the drug U18666A, which inactivates late endosomes and lysosomes, impairs the viral life cycle. The data presented show a clear antiviral effect of two compounds that target the same compartments in different ways. This highlights the relevance of the endosomal–lysosomal compartment for the viral life cycle that should be considered as a target for antivirals.

## 1. Introduction

The Zika virus (ZIKV) is a mosquito-borne flavivirus which was isolated from the blood of a sentinel rhesus monkey in Uganda, in April 1947 [1]. Although serological data indicate a high prevalence of ZIKV in Africa [2], until 2007, only fourteen cases of ZIKV mild illness were reported on the African continent and in Southeast Asia [3,4,5,6,7]. However, this changed after the outbreak on the Yap island, where an estimated 73% of the population was affected [8]. Moreover, during the epidemic in 2014 in French Polynesia, neurological complications such as Guillain–Barré Syndrome (GBS) and newborns with microcephaly manifested aside the typical flu-like symptoms [9,10,11,12]. Shortly thereafter, in 2016, following an increase in microcephaly cases in Brazil in 2015, ZIKV was declared as a public health emergency of international concern (PHEIC) [13]. 

Like other flaviviruses, ZIKV is an enveloped virus that possesses a positive single-stranded RNA genome of approximately 11 kb. The genome encodes the viral polyprotein, which is processed into three structural—capsid/core (C), envelope (E), and precursor of membrane (prM) —and seven non-structural proteins (NS1, NS2A, NS2B, NS3, NS4A, NS4B, and NS5) [14]. The ZIKV life cycle is not yet fully unveiled. The expected life cycle concurs with those of other flaviviruses. The viral life cycle initiates with the interaction between the viral envelope protein and the host cell receptors and adhesion/entry factors, such as DC-SIGN, AXL, Tyro3 and Tim-1 [15]. Nevertheless, there are conflicting data about the relevance of AXL as the primary receptor of ZIKV entry [16,17,18]. After internalization via receptor-mediated endocytosis, it is assumed that the acidification of the late endosomes, also termed multivesicular bodies (MVBs), promotes a structural change in the viral envelope, allowing the fusion between the endosomal membrane and the envelope. This leads to the release of the viral nucleocapsid into the cytoplasm and, consequently, to the disassembling of the capsid and the uncoating of the viral RNA [19,20]. After the genomic RNA has been translated and replicated, a newly synthesized immature viral particle is assembled and conducted to the secretory pathway. In this immature form, the pr peptide covers approximately 12 amino acids of the fusion loop of the envelope protein and, thus, prevents premature fusion within the cell. In the trans-Golgi network (TGN), the viral particle undergoes maturation as a result of a structural modification induced by the low pH that allows the cleavage of the prM by a furin-like protease, exposing the fusion loop. Lastly, the infectious viral particle exits the cell by exocytosis and the pr fragment dissociates from the virion due to the neutral pH of the extracellular environment [21,22].

Besides the bite of an infected mosquito (*Aedes aegypti* and *Aedes albopictus*), ZIKV infection can occur through perinatal transmission, sexual contact and blood transfusion [23,24,25,26]. Since there is no vaccine or a specific antiviral drug available against ZIKV, understanding the viral life cycle and finding ways to control infection has become urgent. Although the ZIKV life cycle is often described to resemble the life cycle of other flavivirus members, a variety of open questions with respect to the viral life cycle still exists. In light of this, the importance of the endosomal–lysosomal compartment for the ZIKV life cycle was investigated. Firstly, the acidification of the late endosomes/MVBs and the TGN compartments was impaired by the highly selective inhibitor of the vacuolar H^+^-ATPase (V-ATPase), bafilomycin A1 [27]. The relevance of the acidification of the endosomal compartment for ZIKV entry has been previously studied in Huh 7 cells by Cortese et al., in JEG-3 cells by Cao et al. and, more recently, in Vero and Cf2Th cells by Persaud et al. [28,29,30]. Our data from A549 cells show that on the one hand, bafilomycin A1 was able to fully inhibit ZIKV entry and, on the other hand, it interferes with viral maturation. Concerning the SH-SY5Y cells, bafilomycin A1 decreased ZIKV entry. In the second part of this study, the functionality of the late endosomes and lysosomes was hindered by the amphipathic steroid drug, U18666A. U18666A inhibits the intracellular trafficking of cholesterol and lipids by the direct inhibition of the Niemann–Pick type C protein. This results in cholesterol accumulation in late endosomes/MVBs and lysosomes, which leads to the impairment of their functionality [31]. Several reports showed that U18666A inhibits crucial steps of dengue virus, hepatitis C virus, Ebola virus and feline coronavirus life cycles [32,33,34,35]. However, no studies were performed to investigate the impact of U18666A on ZIKV infection.

This work provides further insights into the ZIKV life cycle and elucidates crucial steps for viral entry and release.

## 2. Materials and Methods

### 2.1. Cell Culture 

Human epithelial lung carcinoma cells (A549), African green monkey kidney cells (Vero) and human neuroblastoma cells (SH-SY5Y) were grown in Dulbecco’s Modified Eagle’s medium (DMEM) High Glucose (BioWest, Nuaillé, France) supplemented with 10% Fetal Bovine Serum Superior (Biochrom GmbH, Berlin, Germany), 2 mM L-Glutamine (Biochrom GmbH, Berlin, Germany), 100 U/mL penicillin and 100 μg/mL streptomycin (Paul-Ehrlich-Institut facilities, Langen, Germany) in a humidified incubator at 37 °C with 5% CO_2_. Passaging of adherent cells was performed by trypsinization three times a week.

### 2.2. Infection and Treatments

A549 and SH-SY5Y cells were infected with either the French Polynesia PF13/251013-18 or the Uganda 976 strain of ZIKV, which were kindly provided by Dr. Didier Musso from the Institute Louis Malardé, Tahiti, and the European Virus Archive, respectively. In this study, a multiplicity of infection (MOI) of 0.1 was chosen to infect A549 and SH-SY5Y cells for 16 h. The inoculum was removed by washing the cells once with PBS. At the indicated times, cell culture supernatants were collected and stored at –80 °C, while cells were washed once with PBS and then lysed.

Infected A549 cells were treated with 10 nM bafilomycin A1 (Sigma-Aldrich, St. Louis, USA) either 2 h before (pre-) or after (post-) infection. Bafilomycin A1 was permanently present and was renewed at 16, 40, and 64 hours post-infection (hpi) to assure constant levels of the substance in cells. For SH-SY5Y cells, only pre-infection treatment was applied. To investigate the effect of bafilomycin A1 on viral maturation, infected A549 and SH-SY5Y cells were treated with either 50 nM bafilomycin A1 or the respective amount of the vehicle control, DMSO (Genaxxon Bioscience GmbH, Ulm, Germany), during the last 24 h before harvest. Cells and cell culture supernatants were harvested after 72 hpi. To investigate the effect of furin-like protease on viral maturation, infected A549 cells were treated with 10 µM furin inhibitor I (Sigma-Aldrich, St. Louis, USA) during the last 24 h before harvest. Modulation of autophagy was accomplished by 5 mM 3-methyladenine (Selleckchem, Houston, TX, USA) and 100 nM rapamycin (Selleckchem, Houston, USA). A549 cells were treated 2 h after (post-) infection and the treatment was renewed at 16, 40, and 64 hpi to assure constant levels of the compounds in cells. 

Treatment of infected A549 and SH-SY5Y cells with 2 µg/mL U18666A (Sigma-Aldrich, St. Louis, USA) started 2 h after (post-) infection. U18666A was also continuously present and was renewed at 16, 40 and 64 hpi to assure constant levels of the substance in cells. To investigate the effect of U18666A on viral entry, cells were pre-treated for 24 h and then infected for 16 h with either MOI = 0.1 or 1.

### 2.3. Virus Titration

Titration of the virus was performed by plaque forming assay. For this purpose, Vero cells were infected with 100-µL serial dilutions of either cleared cell lysate or supernatant. The cell lysates were achieved by three cycles of freeze–thaw at −80 °C and at 37 °C of the cell suspension, followed by a 10-min centrifugation step at 5000× *g* at 4 °C. At 2 hpi, the inoculum was removed and 0.4% SeaPlaque® agarose (Lonza, Basel, Switzerland) in DMEM complete was poured carefully over the cells. The solidification of the agarose overlay was carried out at room temperature for 15 min. Visualization of the plaques was performed as described in Elgner et al. [36]. The virus titers were expressed in plaque forming units per mL (pfu/mL).

### 2.4. RNA Isolation and cDNA Synthesis

Cells were lysed with peqGOLD TriFast (PEQLAB Biotechnologie GmbH, Erlangen, Germany) and the total intracellular RNA was isolated in accordance with the manufacturer’s instructions. After DNA digestion with RQ1 RNase-free DNase (Promega, Fitchburg, USA), 4 µg of the total RNA was transcribed to cDNA with random hexamer primer and RevertAid H Minus Reverse Transcriptase (Thermo Fischer Scientific, Waltham, USA), as specified by the manufacturer. 

Extracellular RNA was extracted by using the QIAamp Viral RNA Mini Kit (Qiagen, Hilden, Germany), following the manufacturer’s instructions.

### 2.5. RT-qPCR

Quantification of the intracellular ZIKV transcripts was performed by real-time (RT) PCR using the Maxima SYBR Green qPCR Kit (Thermo Fischer Scientific, Waltham, USA) and the following primers: ZIKV-fwd (5’ agatcccggctgaaacactg 3’); ZIKV-rev (5’ ttgcaaggtccatctgtccc 3’); hRPL27-fwd (5’ aaagctgtcatcgtgaagaac 3’), and hRPL27-rev (5’ gctgctactttgcgggggtag 3’). The housekeeping gene human ribosomal protein L27 (hRPL27) was utilized to normalize the amount of intracellular ZIKV transcripts. 

Quantification of the extracellular ZIKV RNA was performed using the LightMix Modular Zika Virus Assay Kit (TIB MOLBIOL, Germany) together with the LightCycler® Multiplex RNA Virus Master Kit (Roche, Basel, Switzerland). 

All quantifications were obtained in the LightCycler® 480 System (Roche, Basel, Switzerland) and according to the manufacturer’s instructions.

### 2.6. Cell Viability

Determination of cell viability after bafilomycin A1, U18666A, and furin inhibitor I treatment was accomplished by using the PrestoBlue® Cell Viability Reagent (Thermo Fischer Scientific, Waltham, USA), as described by the manufacturer. A549 and SH-SY5Y cells were seeded in flat-bottom polystyrene 96-well plates (Greiner, Frickenhausen, Germany), at a density of 1 × 10^4^ cells and 2 × 10^4^ per well, respectively, and treated with different concentrations of the compounds for the desired times. Then, 2% Triton X-100 (Sigma-Aldrich, St. Louis, USA) was included as positive control. The fluorescence of the reagent was measured in the microplate reader Infinite M1000 (Tecan, Basel, Switzerland) after 1 h of incubation at 37 °C.

### 2.7. In Vitro Transcription of ZIKV RLucRNA

Linearization of 40 µg pFLZIKV-RLuc, which was kindly provided by Scott C. Weaver (Institute for Human Infections and Immunity, University of Texas Medical Branch, Galveston, Texas 77555, USA), was achieved by incubation of 40 U ClaI (Thermo Fischer Scientific, Waltham, USA) for 2 h at 37 °C. T7 transcription was performed with 4 µg linearized plasmid using the T7-Scribe™ Standard RNA IVT Kit (Biozym, Hessisch Oldendorf, Germany) for 2 h at 42 °C and subsequent RQ1 RNase-free DNase treatment. After phenol–chloroform extraction, the RNA was dissolved in DEPC water and frozen in 10-µg aliquots at −80 °C.

### 2.8. Electroporation of A549 cells

A549 cells were harvested at a confluency of 80–90%, washed twice with ice-cold PBS and diluted to a final concentration of 5 × 10^6^ cells/mL. For electroporation, 10 μg of in vitro transcribed RNA was mixed with 800 μL cell suspension, added to an electroporation cuvette (4 mm, VWR, Darmstadt, Germany) and immediately pulsed by using a Gene Pulser MXcell™ (BioRad, Dreieich, Germany), which was adjusted to deliver one pulse at 300 V and 950 μF. After incubation for 10 min at room temperature, the electroporated cells were diluted in 12 mL DMEM complete and seeded into appropriate culture dishes. At 4 h post-electroporation, the medium was changed. 

### 2.9. Luciferase Assay

For luciferase reporter-gene assay, stable ZIKV-RLuc producing A549 cells were seeded in polystyrene 6-well plates containing 3 × 10^5^ cells per well (Greiner, Frickenhausen, Germany). The coding sequence for the Renilla luciferase reporter is inserted in the ZIKV genome (ZIKV strain FSS13025, GenBank number KU955593.1). After translation of the polyprotein, the luciferase is released from the polyprotein during the processing.

After the indicated treatments, cells were lysed with 200 µL lysis buffer (pjk, Kleinbittersdorf, Germany), scraped from the plates, transferred to Eppendorf tubes and centrifuged for 5 min at 13,000× *g* at 4 °C. Determination of the luciferase activity was performed using the Gaussia GLOW-Juice Kit (pjk, Kleinbittersdorf, Germany) according to the manufacturer’s instructions. Shortly, 40 µL lysates were analyzed by automated addition of 20 µL Gaussia GLOW-Juice (with CTZ) and subsequent determination of chemiluminescence by an Orion II microplate Luminometer (Titertek, Pforzheim, Germany). Luciferase levels were referred to the protein concentration of the appropriate lysates. Protein amounts were measured using a Bradford Assay, as specified by the manufacturer (Bradford Reagent, Thermo-Scientific, Braunschweig, Germany).

### 2.10. Western Blot

Sample preparation and separation of proteins were performed as described in Glitscher et al. [37]. After separation by SDS-PAGE, proteins were transferred onto a methanol-activated polyvinylidene difluoride (PVDF; 0.45 µm) (Carl Roth, Karlsruhe, Germany) membrane using a semi-dry blotting chamber and a current of 1.3 mA/cm^2^. Proteins of interest were detected with anti-ZIKV NS1 (1:1000; BioFront Technologies, Tallahassee, USA), anti-p62/SQSTM1 (1:1000; Progen Biotechnik GmbH, Heidelberg, Germany), anti-LAMP2 (1:800; BD Biosciences, Franklin Lakes, USA), anti-LC3 (1:1000; BIOZOL Diagnostica Vertrieb GmbH, Echnig, Germany), anti-ZIKV E (1:500; kindly provided by Sami Akhras, Paul-Ehrlich-Institut, Germany) and anti-ZIKV prM (1:1000, GeneTex, Irvine, USA). β-Actin (1:10000; Sigma-Aldrich, St. Louis, USA) detection was used as loading control. As secondary antibodies, either a horseradish peroxidase (HRP)-coupled antibody (GE Healthcare, Little Chalfont, United Kingdom) or a fluorophore-coupled antibody (LI-COR Biosciences, Lincoln, USA) was utilized according to the manufacturer’s instructions. Protein bands were detected with Luminata Forte Western HRP Substrate (Merck Millipore, Darmstadt, Germany) and scientific imaging films (GE Healthcare, Little Chalfont, United Kingdom) or, as alternative, by using the LI-COR Odyssey infrared imaging system (LI-COR Biosciences, Lincoln, USA). Densitometric quantification of the desired proteins was accomplished by Image Studio Lite software (LI-COR Biosciences, Lincoln, USA).

### 2.11. Confocal Laser Scanning Microscopy

Two protocols were used, depending on the primary antibodies and fluorescent dyes utilized. Cells were grown on coverslips and at the desired times, were fixed with either 4% formaldehyde in PBS or with ice-cold ethanol:acetone (1:1) for 20 and 10 min at room temperature, respectively. In case of 4% formaldehyde fixation, cells were permeabilized and blocked as described by Elgner et al. [36]. Cells were incubated with anti-flavivirus group antigen antibody, clone D1-4G2-4-15 (1:300; Merck Millipore, Darmstadt, Germany), anti-ZIKV NS1 (1:1000; BioFront Technologies, Tallahassee, USA), anti-p62/SQSTM1 (1:200; Progen Biotechnik GmbH, Heidelberg, Germany) and anti-lamin A (H-102; 1:500; Santa Cruz Biotechnology, Dallas, USA). Afterwards, cells were incubated with anti-mouse IgG-Alexa 488 (1:10000; Thermo Fisher Scientific, Waltham, USA), anti-guinea pig IgG-Cy3 (1:400, Jackson ImmunoResearch, West Grove, USA), and anti-rabbit IgG-Cy5 (1:400, Jackson ImmunoResearch, West Grove, USA). Together with these secondary antibodies, cells were incubated with the filipin complex (1:100; Sigma-Aldrich, St. Louis, USA) or with 4′,6-diamidino-2-phenylindole (DAPI) (Carl Roth, Karlsruhe, Germany). When cells were fixed with ethanol:acetone, cells were permeabilized and blocked as described by Glitscher et al. [37]. Cells were incubated with anti-flavivirus group antigen antibody, clone D1-4G2-4-15 (1:300; Merck Millipore, Darmstadt, Germany) and anti-p62/SQSTM1 (1:200; Progen Biotechnik GmbH, Heidelberg, Germany), followed by incubation with anti-mouse IgG-Alexa 488 (1:10000; Thermo Fisher Scientific, Waltham, USA) and anti-guinea pig IgG-Cy3 (1:400, Jackson ImmunoResearch, West Grove, USA). Nuclei were visualized with 4DAPI (Carl Roth, Karlsruhe, Germany). In both protocols, the coverslips were mounted on microscope slides with Mowiol and analyzed using the confocal laser scanning microscope LSM 510 Meta and ZEN 2009 software (Carl Zeiss, Oberkochen, Germany).

### 2.12. Transmission Electron Microscopy

For the creation of ultra-thin sections, A549 cells were seeded in culture dishes and treated with 2 µg/mL U18666A (Sigma-Aldrich, St. Louis, USA). After 48 h, the cells were fixed with 2.5% glutaraldehyde (Carl Roth, Karlsruhe, Germany) in DMEM complete for 45 min at room temperature. Afterwards, cells were scrapped off from the culture dish and 2% warmed agarose solution was added to the cells. Then, small agarose blocks containing the cells were cut and post-fixed with 2% osmium tetroxide in PBS. Subsequently, cells were dehydrated with increasing ethanol concentrations and embedded into liquid epoxy resin. The resin was incubated at 60 °C for 48 h for polymerization and cut into ultra-thin sections with an ultramicrotome. These sections were fixed on glow-discharged carbon-coated nickel grids and treated with 2% uranyl acetate for 15 min and subsequent treatment with 2% lead citrate for 5 min. Ultra-thin sections were analyzed by EM-109 transmission electron microscope (Carl Zeiss, Oberkochen, Germany).

### 2.13. Statistical Analysis

Results are described as mean ± standard deviation (SD) from at least 3 independent experiments. The significance of the results was analyzed by Student’s *t* test using GraphPad Prism 7 (GraphPad Software, La Jolla, USA).

## 3. Results

### 3.1. Bafilomycin A1 Inhibits the Establishment of Infection by ZIKV

**Figure 1:**
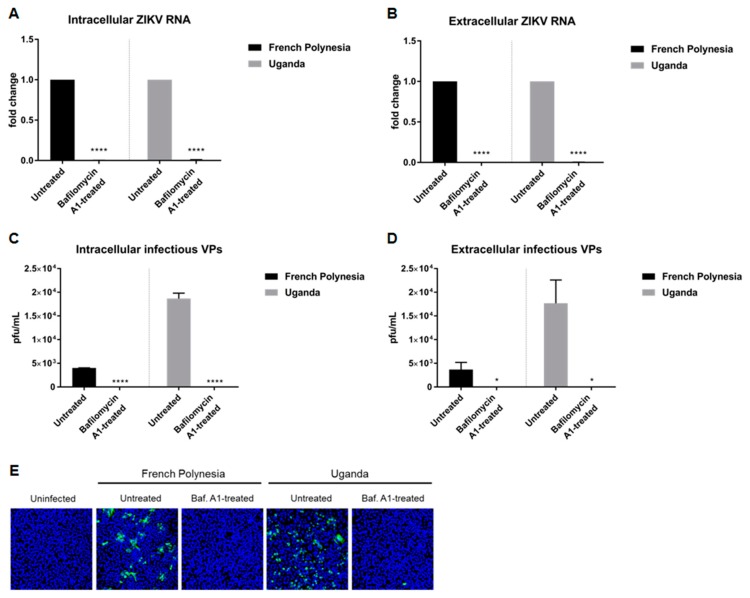
Bafilomycin A1 prevents the establishment of ZIKV infection in A549 cells (**A**) Quantification of the amount of intracellular ZIKV RNA of A549 cells infected with either the French Polynesia or the Uganda strain and treated with 10 nM bafilomycin A1 (pre-infection treatment). Quantification was performed by RT-qPCR and the amount of ZIKV RNA was normalized to the amount of the RPL27 transcripts. Infected untreated cells were used as control; (**B**) Quantification of the amount of extracellular ZIKV RNA of A549 cells infected with either the French Polynesia or the Uganda strain and treated with 10 nM bafilomycin A1 (pre-infection treatment). Quantification was performed by RT-qPCR and infected untreated cells served as control; (**C**) Quantification of the number of intracellular infectious viral particles (VPs) from A549 cells infected with either the French Polynesia or the Uganda strain and treated with 10 nM bafilomycin A1 (pre-infection treatment). Quantification was performed in Vero cells by plaque assay and the number of infectious viral particles is expressed in plaques forming units per mL (pfu/mL). Infected untreated cells were used as control; (**D**) Quantification of the number of extracellular infectious viral particles (VPs) from A549 cells infected with either the French Polynesia or the Uganda strain and treated with 10 nM bafilomycin A1 (pre-infection treatment). Quantification was performed in Vero cells by plaque assay and the number of infectious viral particles is expressed in plaques forming units per mL (pfu/mL). Infected untreated cells served as control; (**E**) A549 cells were infected with either the French Polynesia or the Uganda strain and treated with 10 nM bafilomycin A1 (pre-infection treatment). Cells were fixed with ethanol:acetone (1:1) and nuclei were visualized with DAPI (blue) and the ZIKV envelope protein with a specific antibody (green). Pictures were taken with the 16× objective. * *p ≤* 0.05; **** *p ≤* 0.0001.

Persaud et al. [30] showed that the acidification of the endosomal compartment is crucial for the entry of the ZIKV-MR766 strain. We wondered whether this would differ between a strain responsible for only causing mild symptoms (Uganda 976) and a strain known to be associated with neurological complications (PF13/251013-18). To investigate this, A549 and SH-SY5Y cells were pre-treated with 10 nM bafilomycin A1 and two hours later, in the presence of this substance, the cells were infected using an MOI of 0.1 with either the French Polynesia PF13/251013-18 or the Uganda 976 strain. Analysis of the infection was performed at 24 hours post-infection (hpi) and, in the case of the SH-SY5Y cells, was also analyzed at 48 hpi. The quantification of the number of intra- and extracellular viral genomes by RT-qPCR revealed no significant amount of ZIKV RNA for both strains (Figure 1A,B) in A549 cells. This finding indicates that bafilomycin A1 inhibits the infection process. Quantification of the number of intra- and extracellular infectious particles via plaque assay confirmed this. For both strains, an extreme low or undetectable amount of intracellular and extracellular viral particles was measured (Figure 1C,D). This was further confirmed by confocal laser scanning microscopy of ZIKV-infected cells using an envelope protein-specific antibody. In contrast to the control, no ZIKV-positive cells were observed when treated with bafilomycin A1 (Figure 1E). Concerning the SH-SY5Y cells, the amount of intracellular and extracellular ZIKV RNA was significantly decreased at 24 hpi after bafilomycin A1 treatment. However, unlike for A549, it was still possible to detect a considerable amount of viral RNA (Figure S1A,B). However, confocal laser scanning microscopy of ZIKV-infected cells revealed no ZIKV-positive cells after treatment for both time points analyzed (Figure S1C). The effect of bafilomycin A1 on cell viability in SH-SY5Y cells was investigated by determination of the metabolic activity via PrestoBlue assay. A minor reduction in cell viability could be observed after 24 h after treatment, whereas after 48 h, there was a decrease of approximately 0.4-fold (Figure S2D,E).

Taken together, these data demonstrate that the pre-incubation of ZIKV-permissive cells with bafilomycin A1 completely blocks the establishment of infection in A549 cells and diminishes ZIKV infection in SH-SY5Y cells. Moreover, no major differences were observed between the two strains used. 

### 3.2. Bafilomycin A1 Impedes ZIKV Spread

The above described data show that bafilomycin A1 prevents the establishment of ZIKV infection by inhibition of receptor-mediated endocytosis. To study the effect of this compound on an established infection, A549 cells were primarily infected (MOI = 0.1) and, two hours later, subjected to bafilomycin A1 treatment until harvest. Analysis of the infection was performed at 24, 48, and 72 hpi. 

**Figure 2:**
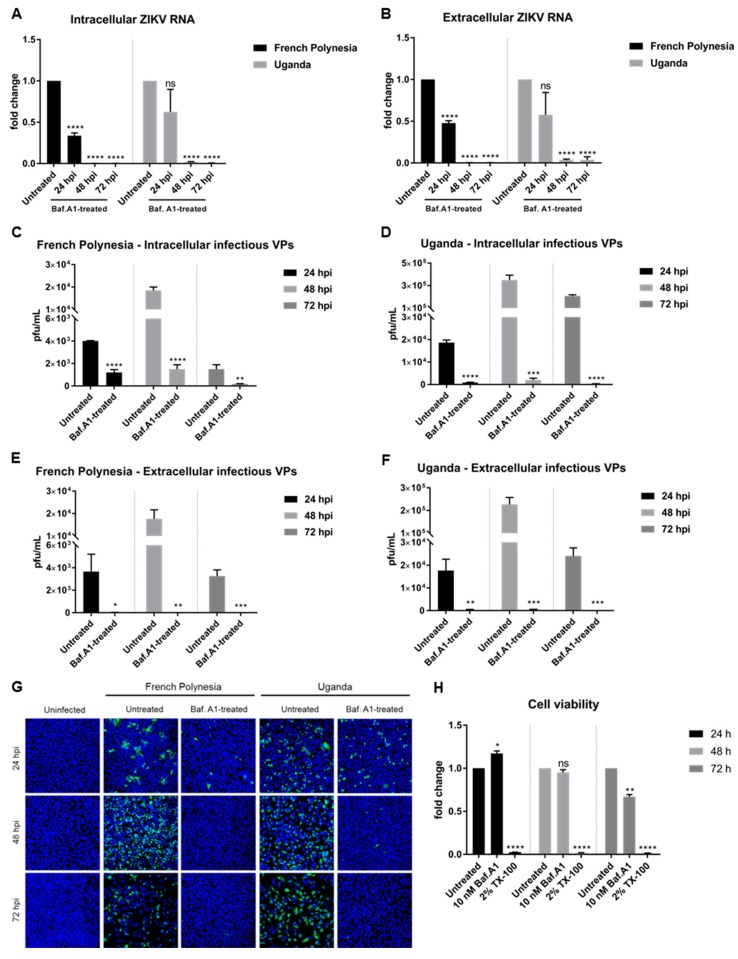
Bafilomycin A1 severely diminishes ZIKV infection in A549 cells. (**A**) Quantification of the amount of intracellular ZIKV RNA of A549 cells infected with either the French Polynesia or the Uganda strain and treated with 10 nM bafilomycin A1 (post-infection treatment) during the indicated times. Quantification was performed by RT-qPCR and the amount of ZIKV RNA was normalized to the amount of the RPL27 transcripts. Infected untreated cells were used as control; (**B**) Quantification of the amount of extracellular ZIKV RNA of A549 cells infected with either the French Polynesia or the Uganda strain and treated with 10 nM bafilomycin A1 (post-infection treatment) during the indicated times. Quantification was performed by RT-qPCR and infected untreated cells served as control; (**C,D**) Quantification of the number of intracellular infectious viral particles (VPs) from A549 cells infected with either the French Polynesia (**C**) or the Uganda (**D**) strain and treated with 10 nM bafilomycin A1 (post-infection treatment) during the indicated times. Quantification was performed in Vero cells by plaque assay and the number of infectious viral particles is expressed in plaques forming units per mL (pfu/mL). Infected untreated cells were used as control; (**E,F**) Quantification of the number of extracellular infectious viral particles (VPs) from A549 cells infected with either the French Polynesia (**E**) or the Uganda (**F**) strain and treated with 10 nM bafilomycin A1 (post-infection treatment) during the indicated times. Quantification was performed in Vero cells by plaque assay and the number of infectious viral particles is expressed in plaques forming units per mL (pfu/mL). Infected untreated cells served as control; (**G**) A549 cells were infected with either the French Polynesia or the Uganda strain and treated with 10 nM bafilomycin A1 (post-infection treatment) during the indicated times. Cells were fixed with ethanol:acetone (1:1) and nuclei were visualized with DAPI (blue) and the ZIKV envelope protein with a specific antibody (green). Pictures were taken with the 16× objective; (**H**) A549 cells were treated with 10 nM bafilomycin A1 during the indicated times and cell viability was quantified by PrestoBlue assay. Cells treated with 2% Triton X-100 (TX-100) served as positive control. Untreated cells were used for normalization. ns = not significant *p >* 0.05; * *p ≤* 0.05; ** *p ≤* 0.01; *** *p ≤* 0.001; **** *p ≤* 0.0001.

Quantification of the amount of intra- and extracellular viral genomes by RT-qPCR showed a reduction in the amount of ZIKV RNA at 24 hpi, diminishing even more at 48 and 72 hpi for both strains (Figure 2A,B). Quantification of the number of intracellular infectious viral particles by plaque assay revealed a significant decrease for both strains. However, a considerable amount of infectious viral particles was still detectable after 24 and 48 hpi when cells were infected with the French Polynesia strain and when compared to the control (Figure 2C,D). In contrast to this, a very strong reduction in the amount of released infectious viral particles was observed after treatment for all three time points analyzed and for both strains (Figure 2E,F). Moreover, confocal laser scanning microscopy showed a lower amount of ZIKV-positive cells when treated with bafilomycin A1 in comparison to the untreated control (Figure 2G).

To control whether the observed effect of bafilomycin A1 on ZIKV-infected cells is not a result of cytotoxicity, the metabolic activity was measured by PrestoBlue assay. Only after 72 h of treatment could a slight reduction be detected (Figure 2H). As the inhibitory effect of bafilomycin A1 on the number of released viral particles and on viral entry is much more pronounced compared to the 0.3-fold decrease in the metabolic activity, it can be assumed that the observed effects of bafilomycin A1 treatment on the viral life cycle are not due to an impact on cellular viability.

Taken together, these results indicate that bafilomycin A1 impairs the spread of ZIKV by the inhibition of de novo infection and by inhibition of the release of infectious viral particles.

### 3.3. Bafilomycin A1 Affects ZIKV Maturation

**Figure 3:**
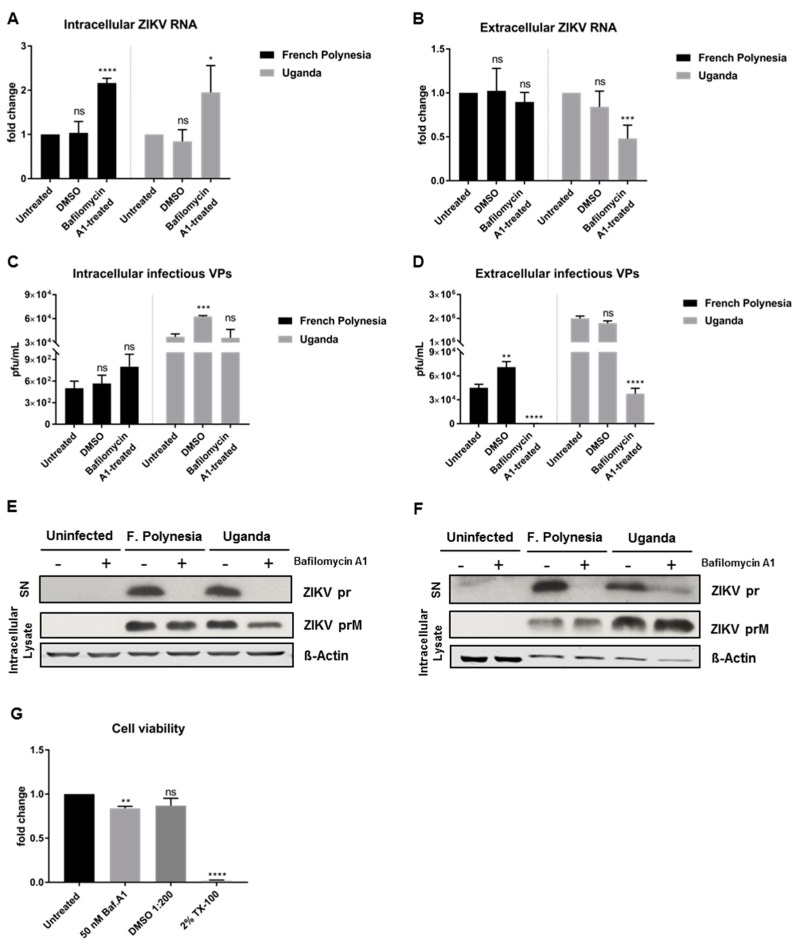
Bafilomycin A1 treatment of ZIKV-infected A549 cells affects the infectiveness of the supernatant. (**A**) Quantification of the amount of intracellular ZIKV RNA of A549 cells infected with either the French Polynesia or the Uganda strain for 72 h and treated with 50 nM bafilomycin A1 (24 h treatment). Quantification was performed by RT-qPCR and the amount of ZIKV RNA was normalized to the amount of the RPL27 transcripts. DMSO was included as vehicle control and infected untreated cells were used as control; (**B**) Quantification of the amount of extracellular ZIKV RNA of A549 cells infected with either the French Polynesia or the Uganda strain for 72 h and treated with 50 nM bafilomycin A1 (24 h treatment). Quantification was performed by RT-qPCR. DMSO was used as vehicle control and infected untreated cells served as control; (**C**) Quantification of the number of intracellular infectious viral particles (VPs) from A549 cells infected with either the French Polynesia or the Uganda strain for 72 h and treated with 50 nM bafilomycin A1 (24 h treatment). Quantification was performed in Vero cells by plaque assay and the number of infectious viral particles is expressed in plaques forming units per mL (pfu/mL). DMSO was used and vehicle control and infected untreated cells served as control; (**D**) Quantification of the number of extracellular infectious viral particles (VPs) from A549 cells infected with either the French Polynesia or the Uganda strain for 72 h and treated with 50 nM bafilomycin A1 (24 h treatment). Quantification was performed in Vero cells by plaque assay and the number of infectious viral particles is expressed in plaques forming units per mL (pfu/mL). DMSO was used as vehicle control and infected untreated cells served as control; (**E**) A549 cells were infected with either the French Polynesia or the Uganda strain and treated with 10 nM bafilomycin A1 (post-infection treatment). The supernatants (used in Section 3.2) and cell lysates were analyzed after 24 hpi by Western blot with a specific antibody against ZIKV-prM/pr peptide. ß-Actin was used as loading control; (**F**) A549 cells were infected with either the French Polynesia or the Uganda strain and treated with 50 nM bafilomycin A1 (post-infection treatment) for 24 h. The supernatants and cell lysates were analyzed after 72 hpi by Western blot with a specific antibody against ZIKV-prM/pr peptide. β-Actin served as loading control; (**G**) A549 cells were treated with 50 nM bafilomycin A1 during 24 h and cell viability was quantified by PrestoBlue assay. Cells treated with DMSO 1:200 and 2% Triton X-100 (TX-100) served as vehicle and positive control, respectively. Untreated cells were used for normalization. ns = not significant *p >* 0.05; * *p ≤* 0.05; ** *p ≤* 0.01; *** *p ≤* 0.001; **** *p ≤* 0.0001.

To enable a clear discrimination between the effect of bafilomycin A1 on viral entry and on viral release, A549 cells were infected with an MOI of 0.1 and only at 48 hpi was the treatment with bafilomycin A1 initiated. At this time point, almost all of the cells (infection reached its peak, as shown in Figure 2G) are infected. The treatment was applied for 24 h and the cell lysates and the respective cell culture supernatants were harvested at 72 hpi. Due to the short duration of the treatment, 50 nM was the concentration chosen for these experiments to ensure the maximum effect of the compound. In these assays, DMSO was included as vehicle control due to the high amount of chemical utilized to perform the experiments. For both strains, treatment with bafilomycin A1 leads to an increase in the amount of intracellular viral genomes, while the number of released viral genomes is significantly reduced for the Uganda strain when compared to the control (Figure 3A,B). Quantification of the number of intracellular infectious viral particles revealed that for both strains bafilomycin A1 does not lead to a significant change, although analysis of the supernatant unveiled a strong inhibition of the release of viral particles (Figure 3C,D). To further investigate the effect of bafilomycin A1 on the ZIKV maturation, cell culture supernatants were analyzed by Western blot and the prM protein/pr peptide was detected. Analysis of the cell culture supernatants harvested at 24 hpi from Section 3.2 (Figure 2E,F) revealed that the pr peptide could not be detected when the infected cells were treated with bafilomycin A1 (Figure 3E). Furthermore, the amount of intracellular ZIKV prM slightly decreased after treatment (Figure 3E). Moreover, the cell culture supernatants harvested at 72 hpi confirmed the same results as in the ones shown in Figure 3C,D. For the French Polynesia strain, a hardly detectable amount of the pr peptide was detected, while for the Uganda strain, a strong reduction in the amount of pr peptide could be observed (Figure 3F). Regarding the amount of intracellular ZIKV prM, no significant changes were visible for the French Polynesia strain, whereas for the Uganda strain a small accumulation could be observed after bafilomycin A1 treatment (Figure 3F). The effect of 50 nM bafilomycin A1 on cell viability was measured by PrestoBlue assay to control the specificity of the observed results. Although a minor decrease in cell viability was detected after treatment, the effect on infectiveness is much more pronounced than this reduction (Figure 3G).

The same study was investigated in SH-SY5Y cells. However, a fully established infection could not be accomplished at 48 hpi. Quantification of the amount of intra- and extracellular ZIKV genomes shows a considerable reduction in ZIKV RNA after bafilomycin A1 treatment (Figure S2A–C). 

These data show that treatment of ZIKV-infected A549 cells with bafilomycin A1 affects the infectiveness of the supernatant.

**Figure 4:**
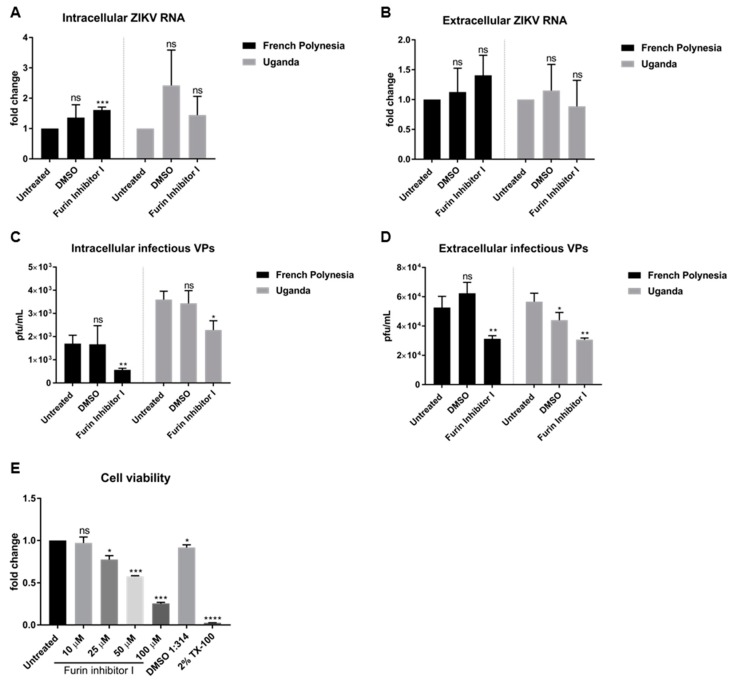
Furin inhibitor I diminishes ZIKV infectivity in A549 cells. (**A**) Quantification of the amount of intracellular ZIKV RNA of A549 cells infected with either the French Polynesia or the Uganda strain for 72 h and treated with 10 µM furin inhibitor I (24 h treatment). Quantification was performed by RT-qPCR and the amount of ZIKV RNA was normalized to the amount of the RPL27 transcripts. DMSO was included as vehicle control and infected untreated cells were used as control; (**B**) Quantification of the amount of extracellular ZIKV RNA of A549 cells infected with either the French Polynesia or the Uganda strain for 72 h and treated with 10 µM furin inhibitor I (24 h treatment). Quantification was performed by RT-qPCR. DMSO was used as vehicle control and infected untreated cells served as control; (**C**) Quantification of the number of intracellular infectious viral particles (VPs) from A549 cells infected with either the French Polynesia or the Uganda strain for 72 h and treated with 10 µM furin inhibitor I (24 h treatment). Quantification was performed in Vero cells by plaque assay and the number of infectious viral particles is expressed in plaques forming units per mL (pfu/mL). DMSO was used and vehicle control and infected untreated cells served as control; (**D**) Quantification of the number of extracellular infectious viral particles (VPs) from A549 cells infected with either the French Polynesia or the Uganda strain for 72 h and treated with 10 µM furin inhibitor I (24 h treatment). Quantification was performed in Vero cells by plaque assay and the number of infectious viral particles is expressed in plaques forming units per mL (pfu/mL). DMSO was used as vehicle control and infected untreated cells served as control; (**E**) A549 cells were treated with different concentrations of furin inhibitor I during 24 h and cell viability was quantified by PrestoBlue assay. Cells treated with DMSO 1:314 and 2% Triton X-100 (TX-100) served as vehicle control for the chosen concentration and positive control, respectively. Untreated cells were used for normalization. ns = not significant *p >* 0.05; * *p ≤* 0.05; ** *p ≤* 0.01; *** *p ≤* 0.001; **** *p ≤* 0.0001.

To investigate whether the loss of the infectiveness of ZIKV particles after bafilomycin A1 treatment is due to impaired viral maturation, A549 cells were infected with an MOI of 0.1 and, at 48 hpi, were treated with 10 µM furin inhibitor I, a known inhibitor of viral maturation. Analysis of the infection was performed at 72 hpi. The quantification of the number of intra- and extracellular viral RNA by RT-qPCR revealed that furin treatment does not affect the number of intra- or extracellular viral genomes. No significant differences of ZIKV RNA for both strains, with the exception of a slight accumulation of the amount of intracellular genomes for the French Polynesia strain after furin inhibitor I treatment (Figure 4A,B) were observed.

Moreover, the number of intra- and extracellular infectious viral particles was determined by plaque assay. In contrast to the quantification of the viral genomes, a significant decrease in the number of intra- and extracellular infectious viral particles was observed for both strains after furin inhibitor I treatment (Figure 4C,D). To be certain that the concentration chosen to treat the cells was not cytotoxic, the metabolic activity after different concentrations of furin inhibitor I was measured by PrestoBlue assay. From all the concentrations analyzed, only 10 µM did not affect cell viability.

Taken together, the results indicate that interference with furin activity by furin inhibitor I affects ZIKV maturation.

### 3.4. Effect of Bafilomycin A1 on ZIKV Infection Is Not Due to Inhibition of Autophagy

**Figure 5:**
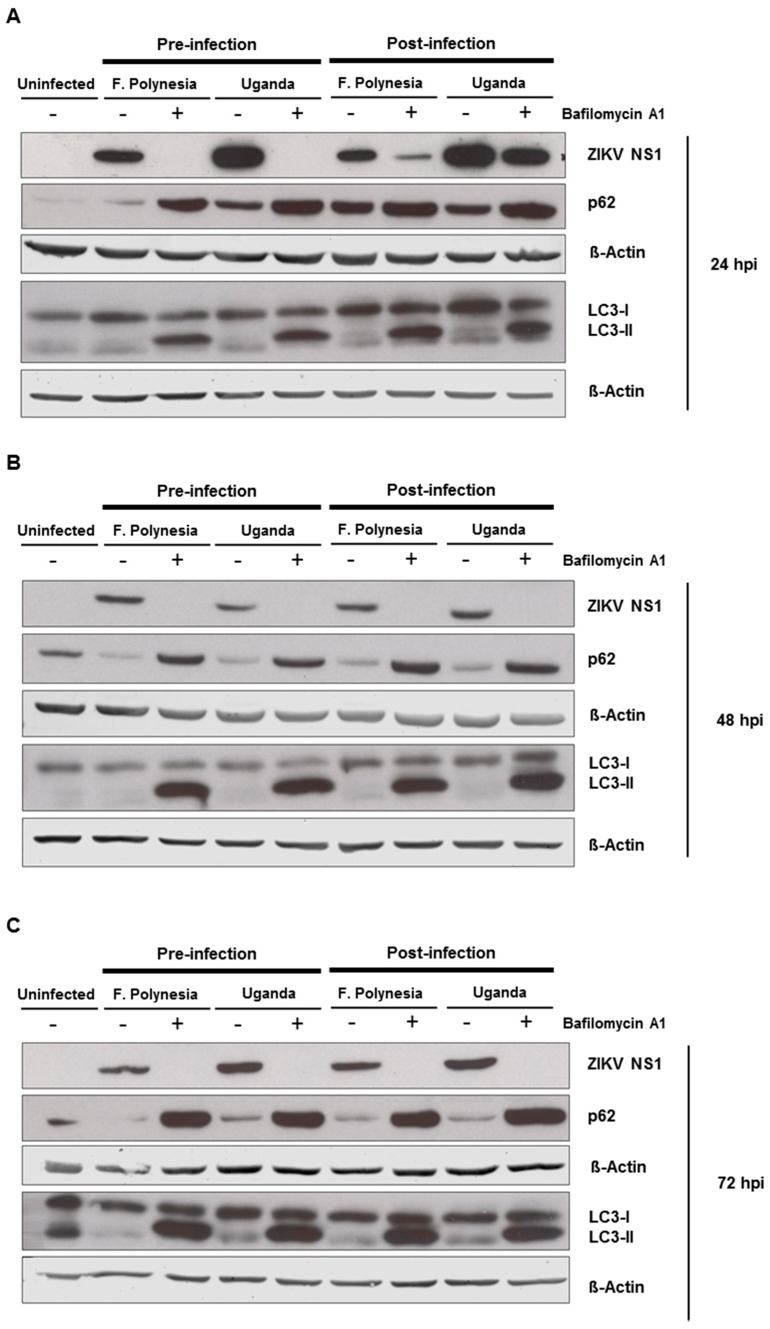

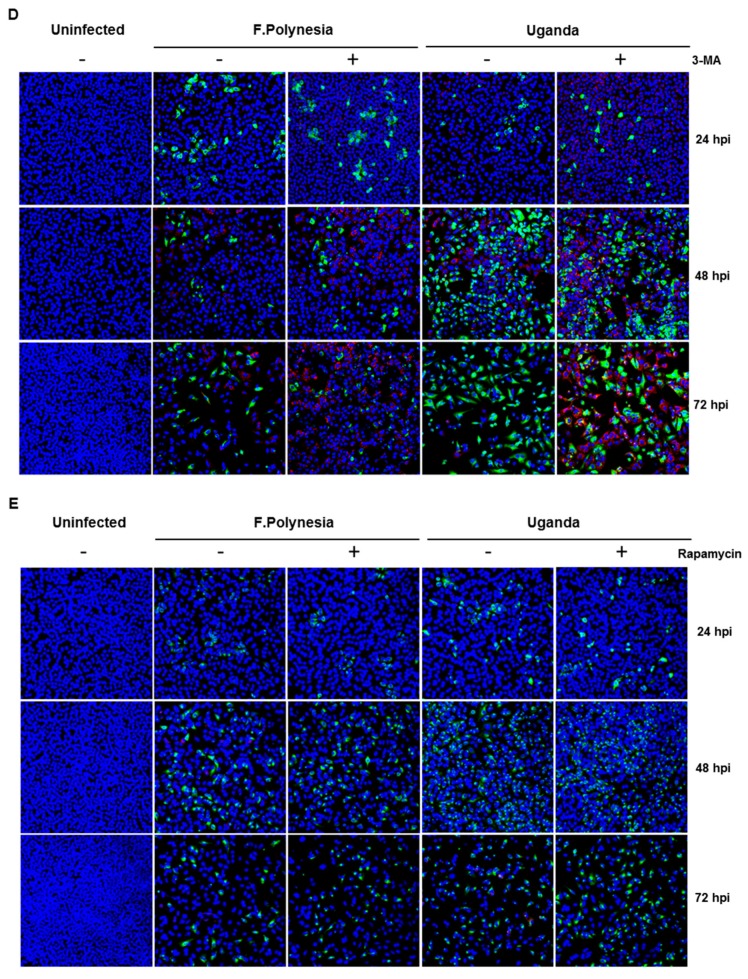
Autophagy modulation in ZIKV-infected cells. (**A**–**C**) A549 cells were infected with either the French Polynesia or the Uganda strain and treated with 10 nM bafilomycin A1 (pre- and post-infection treatment) during 24 (**A**), 48 (**B**), and 72 (**C**) hpi. Cell lysates of the indicated times were analyzed by Western blot with specific antibodies against the ZIKV NS1, p62, LC3, and β-Actin. The latest was included as loading control; (**D**,**E**) A549 cells were infected with either the French Polynesia or the Uganda strain and treated with either 5 mM 3-MA (**D**) or 100 nM rapamycin (**E**) (post-infection treatment) during the indicated times. Cells were fixed with ethanol:acetone (1:1) and analyzed by confocal laser scanning microscopy. Nuclei were visualized with a DAPI (blue) and the ZIKV envelope protein (green) and p62 (red) with specific antibodies.

Since flaviviruses can exploit the autophagic machinery to enhance its infectivity and bafilomycin A1 is a well-known autophagy inhibitor, we investigated whether in our system bafilomycin A1 affects the ZIKV-dependent induction of autophagy. For this purpose, A549 cells were subjected to pre-infection and post-infection treatment. Analysis of the infection and the amount of autophagy proteins was performed at 24, 48, and 72 hpi by Western blot analysis. For all time points analyzed, an accumulation of p62 and LC3-II proteins could be detected. The inhibition of ZIKV infection was controlled by the amount of ZIKV NS1 (Figure 5A–C). This indicates that the chosen concentration of bafilomycin A1 has an impact on autophagy.

To investigate whether the observed effects of bafilomycin A1 are mainly due to its effect on autophagy, A549 cells were either treated with 5 mM the autophagy inhibitor 3-methyladenine (3-MA) or 100 nM rapamycin that acts as an inducer of autophagy. The number of infected cells was analyzed by confocal laser scanning microscopy using an envelope protein-specific antibody. The fluorescence microscopy revealed that the modulation of autophagy in A549 cells does not affect the number of ZIKV-positive cells (Figure 5D,E). These data suggest that the observed effects of bafilomycin A1 treatment are not primarily due to an effect on autophagy.

### 3.5. Cholesterol Accumulation in Late Endosomes and Lysosomes Impairs ZIKV Infection

To further characterize the relevance of the endosomal–lysosomal compartment, A549 cells were treated with 2 µg/mL U18666A (treatment was applied 2 h after infection). U18666A-treated cells accumulate cholesterol in late endosomes/MVBs and lysosomes, which results in the inhibition of their activity. Cells were infected with an MOI of 0.1 with either the French Polynesia PF13/251013-18 or the Uganda 976 strain and infection was analyzed at 24, 48, and 72 hpi.

**Figure 6:**
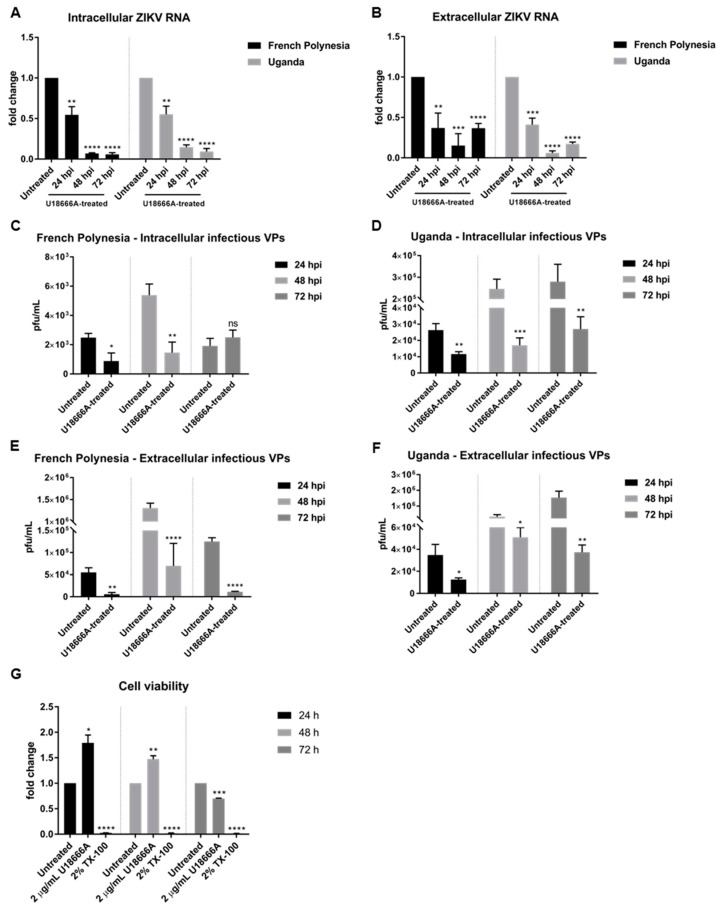
U18666A decreases ZIKV infection in A549 cells. (**A**) Quantification of the amount of intracellular ZIKV RNA of A549 cells infected with either the French Polynesia or the Uganda strain and treated with 2 µg/mL U18666A (post-infection treatment) during the indicated times. Quantification was performed by RT-qPCR and the amount of ZIKV RNA was normalized to the amount of the RPL27 transcripts. Infected untreated cells were used as control; (**B**) Quantification of the amount of extracellular ZIKV RNA of A549 cells infected with either the French Polynesia or the Uganda strain and treated with 2 µg/mL U18666A (post-infection treatment) during the indicated times. Quantification was performed by RT-qPCR and infected untreated cells served as control; (**C,D**) Quantification of the number of intracellular infectious viral particles (VPs) from A549 cells infected with either the French Polynesia (**C**) or the Uganda (**D**) strain and treated with 2 µg/mL U18666A (post-infection treatment) during the indicated times. Quantification was performed in Vero cells by plaque assay and the number of infectious viral particles is expressed in plaques forming units per mL (pfu/mL). Infected untreated cells were used as control; (**E,F**) Quantification of the number of extracellular infectious viral particles (VPs) from A549 cells infected with either the French Polynesia (**E**) or the Uganda (**F**) strain and treated with 2 µg/mL U18666A (post-infection treatment) during the indicated times. Quantification was performed in Vero cells by plaque assay and the number of infectious viral particles is expressed in plaques forming units per mL (pfu/mL). Infected untreated cells served as control; (**G**) A549 cells were treated with 2 µg/mL U18666A during the indicated times and cell viability was quantified by PrestoBlue assay. Cells treated with 2% Triton X-100 (TX-100) served as positive control. Untreated cells were used for normalization. ns = not significant *p >* 0.05; * *p ≤* 0.05; ** *p ≤* 0.01; *** *p ≤* 0.001; **** *p ≤* 0.0001.

The quantification of the number of intra- and extracellular viral genomes via RT-qPCR showed a significant reduction in the amount of ZIKV RNA for both strains (Figure 6A,B). Comparable results were obtained for ZIKV-infected SH-SY5Y cells (Figure S3A–C). Moreover, this effect was confirmed by quantification of the number of intra- and extracellular infectious viral particles by plaque assay. Infected cells that were subjected to treatment presented significantly lower virus titers, regardless of the strain used for infection (Figure 6C–F). Nevertheless, after 72 hpi the intracellular virus titer for the French Polynesia infected cells did not significantly differ from the U18666A-treated cells. A possible explanation could be that at this time point only a low production of viral particles occurs due to the cytopathic effect in infected untreated cells, which results in the release of viral particles from the dead cells. In the U18666A-treated cells, the infection is attenuated and therefore, a less pronounced cytopathic effect occurs. Both effects together contribute to the absence of a significant impact of U18666A at this time point.

To control whether the observed effect of U18666A on ZIKV-infected cells is specific and not due to impaired cellular viability, the metabolic activity was measured by PrestoBlue assay. Similar to bafilomycin A1, only after 72 h of treatment could a minor reduction be detected (Figure 6G). Since the inhibitory effect of U18666A could already be observed at earlier time points, it can be assumed that the effects are not based on cell toxicity. 

**Figure 7:**
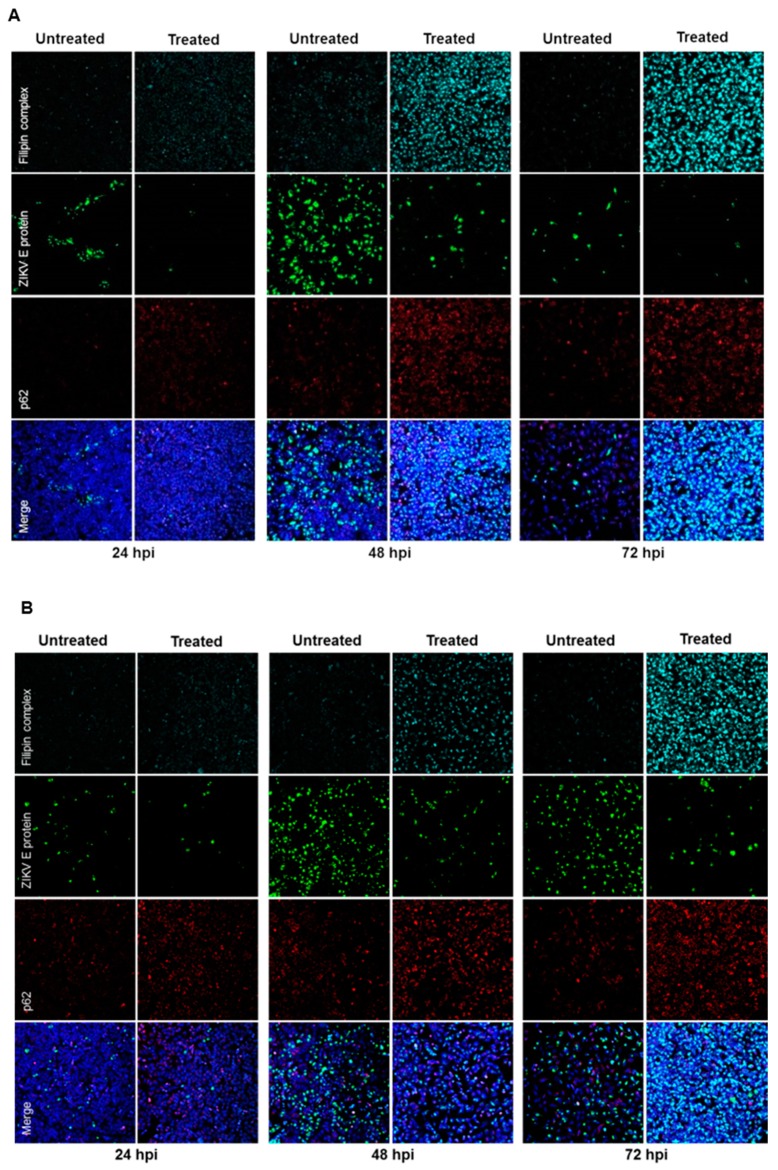

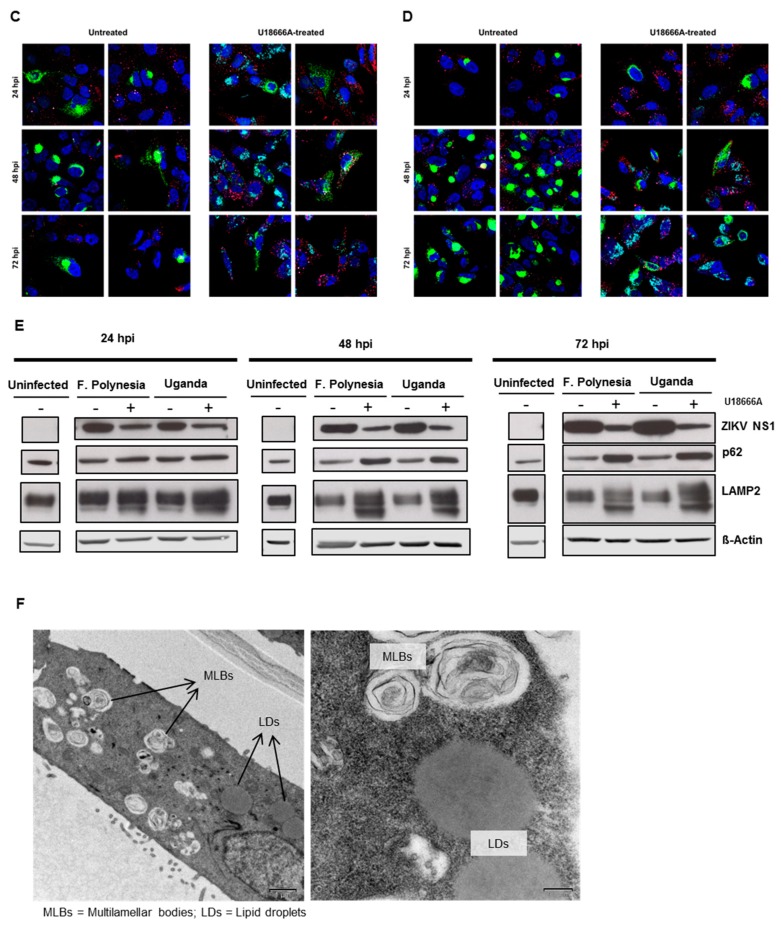
U18666A affects the amount and the intracellular localization of ZIKV proteins in A549 cells. (**A**–**D**) A549 cells were infected with either the French Polynesia (**A**,**C**) or the Uganda (**B**,**D**) strain and treated with 2 µg/mL of U18666A (post-infection treatment) during the indicated times. Cells were fixed with 4% formaldehyde and analyzed by confocal laser scanning microscopy. Nuclei were visualized with a lamin A-specific antibody (blue), the ZIKV envelope protein with a specific antibody (green), p62 with a specific antibody (red) and free cholesterol with the filipin complex dye (cyan); Pictures were taken with either the 16× (**A**,**B**) or 100× objective (**C**,**D**); (**E**) A549 cells were infected with either the French Polynesia or the Uganda strain and treated with 2 µg/mL of U18666A (post-infection treatment). Cell lysates of the indicated times were analyzed by Western blot with specific antibodies against the ZIKV NS1, p62, LAMP2, and β-Actin. The latest was included as loading control; (**F**) Transmission electron microscopy images of U18666A-treated cells.

Furthermore, the effect of U18666A on ZIKV-infected cells was analyzed by confocal laser scanning microscopy. A gradual accumulation of cholesterol was monitored by the dye filipin complex that binds to free cholesterol. Moreover, the impaired lysosomal functionality, as a consequence of the U18666A treatment, was verified by an increased amount of the autophagy substrate, p62. Although antiviral activity was already visible at 24 hpi, characterized by the lower number of ZIKV-positive cells after treatment, the maximum of cholesterol accumulation was observed at 72 hpi (Figure 7A,B). In addition, the same samples were analyzed at a higher magnification to investigate whether the treatment could influence the intracellular localization and distribution of the E protein. After U18666A treatment, an evident change in the subcellular distribution and amount in the cell of the envelope protein (green signal) could be visualized (Figure 7C,D). In U18666A-treated cells, most of the intensively stained large green structures disappeared and a more dispersed staining could be observed. Besides the clear impact on the amount and distribution of the structural protein E, the effect of the treatment on the amount of a non-structural protein (NS1) was investigated by Western blot analysis. The amount of NS1 was reduced after U18666A treatment for both strains and at all time points investigated, but especially more pronounced after 48 and 72 hpi. This is in accordance with the RNA data shown in Figure 6A,B. The efficacy of the treatment was reflected by the increase in the amount of the lysosome-associated protein 2 (LAMP2) and p62 (Figure 7E). In addition, the functionality of the inhibitor was reflected by the formation of multilamellar bodies (MLBs) and lipid droplets as evidenced by transmission electron microscopy of ultra-thin sections of U18666A-treated cells (Figure 7F). 

Taken together, U18666A displays antiviral activity against ZIKV as a result of a defective functionality of endosomes and lysosomes. 

### 3.6. ZIKV Replication Is Affected by U18666A

**Figure 8:**
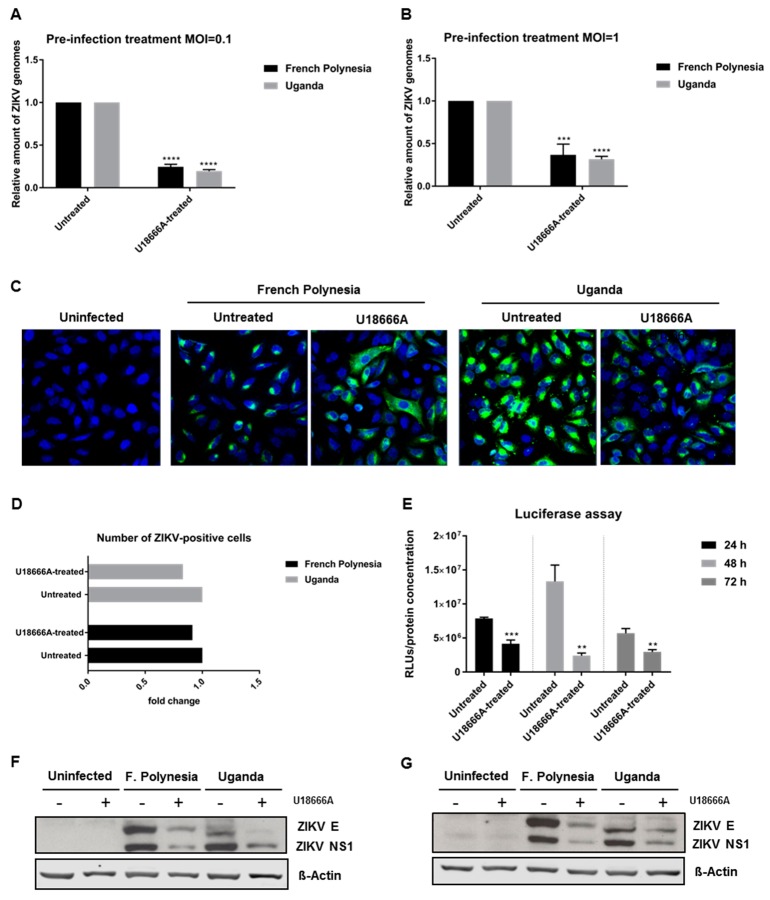
U18666A affects ZIKV replication in A549 cells. (**A**) Quantification of the amount of intracellular ZIKV RNA of A549 cells infected with an MOI = 0.1 after 16 hpi with either the French Polynesia or the Uganda strain and pre-treated with 2 µg/mL U18666A (pre-infection treatment) for 24 h. Quantification was performed by RT-qPCR and the amount of ZIKV RNA was normalized to the amount of the RPL27 transcripts. Infected untreated cells were used as control; (**B**) Quantification of the amount of intracellular ZIKV RNA of A549 cells infected with an MOI = 1 after 16 hpi with either the French Polynesia or the Uganda strain and pre-treated with 2 µg/mL U18666A (pre-infection treatment) for 24 h. Quantification was performed by RT-qPCR and the amount of ZIKV RNA was normalized to the amount of the RPL27 transcripts. Infected untreated cells were used as control; (**C**) A549 cells were infected with an MOI = 1 with either the French Polynesia or the Uganda strain and pre-treated with 2 µg/mL U18666A (pre-infection treatment) for 24 h. Cells were fixed with 4% formaldehyde after 16 hpi and analyzed by confocal laser scanning microscopy. Nuclei were visualized with a lamin A-specific antibody (blue) and the ZIKV envelope protein with a specific antibody (green). Pictures were taken with the 40× objective. (**D**) Quantification of the number of ZIKV-positive cells from Figure 8C. Quantification is referent to at least 100 cells from each condition; (**E**) Luciferase reported gene assay of ZIKV-replicating A549 cells after 24, 48 and 72 h treatment with 2 µg/mL U18666A; (**F,G**) Western blot analysis of A549 cells that were infected with an MOI = 0.1 (**F**) and MOI = 1 (**G**) with either the French Polynesia or the Uganda strain and pre-treated with 2 µg/mL U18666A (pre-infection treatment) for 24 h. Infection was detected by ZIKV E and NS1 specific antibodies. ** *p ≤* 0.01; *** *p ≤* 0.001; **** *p ≤* 0.0001.

To investigate which steps of the viral life cycle are affected by U18666A, A549 cells were pre-treated with 2 µg/mL U18666A for 24 h and infected with either an MOI = 0.1 or 1 in the presence of this substance. Infection with either the French Polynesia PF13/251013-18 or the Uganda 976 strain was analyzed after 16 hpi by RT-qPCR. The quantification of the number of intracellular viral genomes showed a 0.7-fold reduction for both strains, regardless of the amount of viral input used (Figure 8A,B). Moreover, to discriminate whether this decrease was based on impaired viral entry or viral replication, A549 cells were subjected to the same conditions as described above and analyzed by confocal laser scanning microscopy. Here, cells were exclusively infected with an MOI = 1. Similar to what was observed before in U18666A-treated cells, a dispersion of the ZIKV-envelope protein was observed (Figure 8C). Even though the signal intensity for the envelope protein was reduced and the distribution of the envelope protein was changed under these conditions, the total number of ZIKV-positive cells was not significantly affected (Figure 8D). To further corroborate these data, reporter gene assays were performed. ZIKV-RLuc producing A549 cells were treated with 2 μg/mL U18666A for 24, 48 and 72 h. In these cells, the luciferase activity directly reflects the genome replication. The reporter gene assay revealed a significant decrease in the luciferase activity in U18666A-treated cells (Figure 8E). In addition, Western blot analyses of cellular lysates derived from U18666A-treated cells were performed using ZIKV envelope- and ZIKV NS1-specific antibodies. The blots and the corresponding quantification indicate that the amount of ZIKV NS1 and ZIKV envelope protein is reduced in U18666A-treated cells (Figure 8F,G and Figure S4).

Taken together, these data show that U18666A inhibits ZIKV replication.

## 4. Discussion

The ZIKV entry process is not fully understood. Bafilomycin A1 is a well-characterized inhibitor of the V-ATPase and it has been frequently used to restrain the infection of viruses internalized by receptor-mediated endocytosis. Persaud et al. [30] demonstrate that this compound inhibits the entry of ZIKV MR766 strain in Vero and Cf2Th cells. Our data show that bafilomycin A1 strongly impairs the entry of ZIKV Uganda 976 (known to cause only mild symptoms) and ZIKV French Polynesia PF13/251013-18 (associated with neurological complications) in A549 and in SH-SY5Y cells. These data are in line with previous observations describing that ammonium chloride and chloroquine have the potential to block ZIKV infection [38,39,40]. It also reflects the observations that the infection process of a variety of other flaviviruses, such as dengue virus or West Nile virus, can be inhibited by preventing the receptor-mediated endocytosis.

When bafilomycin A1 was added post-infection, there was a reduction in the number of released viral genomes after 24 hpi and a complete inhibition after 48 and 72 hpi. However, the amount of released infectious viral particles for all time points was practically zero. This indicates that, in addition to its inhibitory effect on the entry process, bafilomycin A1 also affects the release of viral particles. At 24 hpi, the presence of viral genomes in the supernatant of bafilomycin A1-treated cells, but no significant amount of infectious viral particles suggests that the genomes measured represent defective/unprocessed virons or naked capsids/genomes. The observed inhibitory effect in our experiments seems to be in contrast to data published by Cortese et al. [28] In this study, no inhibitory effect of bafilomycin A1 treatment was observed when this substance was applied 3 h post-infection. However, the concentration of 2.5 nM used in this study could be below the concentration required for an efficient inhibition of the V-ATPase that is described with 10 nM [41]. Moreover, there is a difference in the cell culture model. In the study of Cortese et al., Huh7 cells were used and they might differ in their capacity to metabolize xenobiotics. The inhibitory effect of bafilomycin A1 on the release of ZIKV was further confirmed on a fully established infection after 48 hpi. Once more, the number of released infectious viral particles was strongly decreased, while again the amount of released viral RNA was less affected, which reflects the release of non-infectious particles or naked genomes. During ZIKV-morphogenesis, processing of the prM membrane protein by a furin-like protease occurs, as described for a variety of other flaviviruses [21,22,42]. Changing the pH of the TGN compartment by bafilomycin A1 strongly impairs the activity of this enzyme and could affect the conformational changes required for proteolytic processing. Indeed, Western blot analysis revealed that the formation of the pr-peptide, which reflects the processing of the prM protein, is prevented by bafilomycin A1. The quantification of the released viral genomes indicates that there is still a significant release of viral genomes in case of the cells treated with 50 nM bafilomycin A1 for 24 h before harvest (Figure 3B). We interpret this as the release per se is not strongly affected and, in accordance with this, no strong intracellular accumulation of prM is observed (Figure 3F). However, plaque assay data reveal that the infectivity of the released particles is strongly decreased (Figure 3D). We hypothesize that this could be due to an impaired processing of the prM to pr fragment that is reflected by the Western blot analysis shown in Figure 3F. The specificity of the observed effects was controlled by a viability assay that showed only a moderate reduction in the metabolic activity at 72 h. This decrease of approximately 30% is much less pronounced as compared to the almost complete loss of viral RNA and infectious viral particles.

The data from the furin inhibitor I experiments show that the inhibition of the furin activity leads to a reduced amount of released infectious viral particles which could support this hypothesis. It should be noted that in order to avoid the cytotoxic effects of the furin inhibitor I, this compound was used at a concentration of 10 μM which is significantly lower than the concentration of 50 μM that is required for a complete inhibition of the furin activity [43]. 

As bafilomycin A1 is known to inhibit autophagy, we investigated whether there is an interference of bafilomycin A1 with the ZIKV-dependent induction of autophagy in our experimental system and whether this is relevant for the observed bafilomycin A1-dependent effects described here. Indeed, we observed that bafilomycinA1 impairs the ZIKV-dependent induction of autophagy. However, modulation of autophagy either by 3-MA or by rapamycin had no effect on the number of infected cells. This suggests that the bafilomycin-dependent interference with autophagy is not primarily causative for the observed effects in our study. This seems to be in contrast to studies that describe a direct effect of autophagy modulation on the ZIKV life cycle, but the experimental settings, including the incubation periods and concentrations, were different from our study [15,29,44].

To further gain insight on the relevance of the endosomal–lysosomal compartment, the intracellular cholesterol transport inhibitor U18666A was used. In this study, the effect of U18666A on the ZIKV life cycle was investigated for the first time. Prolonged U18666A treatment results in the inhibition of the functionality of late endosomes/MVBs and lysosomes, as a consequence of the accumulation of cholesterol in these organelles [31]. This was experimentally verified by the increased number of lipid droplets and the formation of non-functional lysosomes, the so-called multilamellar bodies (MLBs). Although U18666A could significantly diminish the amount of intracellular and extracellular viral genomes and infectious viral particles for all time points analyzed, the infection persisted until 72 hpi. Nevertheless, at 72 hpi, the number of intracellular infectious viral particles in French Polynesia-infected cells was approximately the same as when treated with U18666A. This can be explained by the fact that this strain has a higher cytopathic effect and causes significant cell death after 48 hpi and before the wash at 64 hpi. Thus, the virus titers are very similar to the ones measured at 24 hpi. Since the treatment slows down, the typical infection kinetic, a higher number of intracellular infectious viral particles could be detected. The persistence of ZIKV infection until 72 hpi after U18666A treatment could be easily observed by confocal laser scanning microscopy analysis, where ZIKV-positive cells were still found at this time point. Even in the presence of the U18666A treatment, the number of ZIKV-positive cells increased from 24 to 48 hpi. These findings might indicate that the main effect is on viral replication. However, from these experiments, we could not exclude completely that this compound does not have a minor effect on viral entry. Moreover, the intracellular localization and distribution of the ZIKV envelope protein also changed after treatment. In U18666A-treated cells, the viral envelope protein was found dispersed throughout the cytoplasm instead of concentrated in this dot-like compartment. These data support the possible effect of U18666A on viral replication. 

Nevertheless, no definitive conclusion can be drawn from these experiments since the treatment started 2 h after infection and the cholesterol accumulation can be visible only after 16 h of treatment as previously reported in Poh et al. and Takano et al. [32,35]. To clarify this point, infected cells (MOI = 0.1 or 1) were pre-treated for 24 h and analyzed by RT-qPCR. Different amounts of viral input led to the same fold-reduction (0.7) after 16 hpi, suggesting that the effect of U18666A might be on viral replication. These findings were further confirmed by confocal laser scanning microscopy, where a considerable number of ZIKV-positive cells could be observed after pre-treatment with U18666A, and by analysis of the replication of a luciferase reporter virus. If the effect was on viral entry, it would only be possible to observe, in each cell, a single viral particle and not a significant amount and distribution of the envelope protein, as described by Poh et al.—U18666A trapped dengue virus in late endosomes [32]. Interestingly, for other flaviviruses, such as for hepatitis C virus, Elgner et al. showed that the replication was not affected by U18666A treatment as the number of intracellular viral genomes and the amount of NS3 and NS5A remained constant [33]. Here, we also investigated the effect of U18666A on the amount of a non-structural protein (NS1) and the envelope protein. Western blot analysis showed that, the amount of NS1 and E protein was decreased after U18666A treatment. Once more, the data strongly points towards an effect at the replication level. The inhibition of the trafficking of intracellular cholesterol by U18666A and its accumulation in late endosomes and lysosomes has repercussions in the membrane composition and fluidity of the endoplasmic reticulum and therefore, most likely affects the formation of the replication complex. There are reports describing the impact of U18666A on the replication of flaviviruses, such as HCV and DENV. Although there are differences in the structural organization of the replication factories and the formation of a membranous web structure, there seems to be parallels that make them accessible for an inhibition by U18666A [45,46,47].

In light of the fact that nowadays neither a vaccine nor a specific therapy for ZIKV exists, substances interfering with the acidification and functionality of the endosomal–lysosomal compartment and with the intracellular transport of cholesterol could be a helpful first-line treatment to prevent the spread of ZIKV infection in an epidemic situation.

## 5. Conclusions

We conclude from these data that bafilomycin A1 exerts an inhibitory effect on the ZIKV life cycle by interfering with the entry and the release processes. Moreover, we conclude that the steroid drug U18666A affects the ZIKV life cycle by inhibiting replication.

## Supplementary Materials

The following are available online at https://www.mdpi.com/1999-4915/11/6/524/s1, Figure S1: Bafilomycin A1 decreases ZIKV entry in SH-SY5Y cells, Figure S2: Bafilomycin A1 diminishes ZIKV infection in SH-SY5Y cells, Figure S3: U18666A inhibits ZIKV infection in SH-SY5Y cells, Figure S4: U18666A decreases the amount of ZIKV E and NS1 proteins in A549 cells.
