# Supplementary material for: Bafilomycin A1 and U18666A Efficiently Impair ZIKV Infection
Source: Viruses. 2019 Jun 6;11(6):524. doi: 10.3390/v11060524 (PMC6630673; doi:10.3390/v11060524)
Supplement: Supplementary file 1 [file viruses-11-00524-s001.pdf]

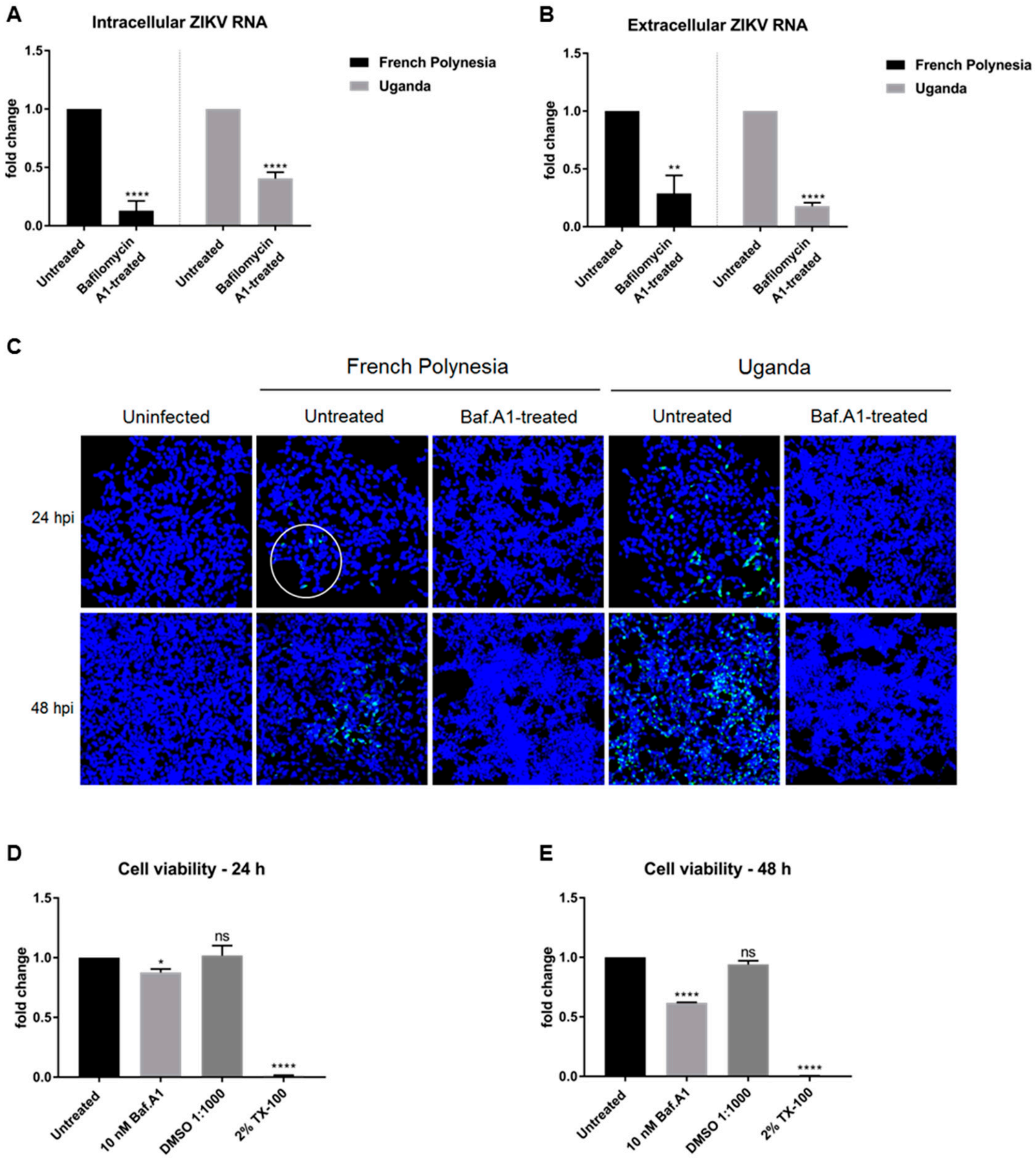

Figure S1. Bafilomycin A1 decreases ZIKV entry in SH-SY5Y cells (**A**) Quantification of the amount of intracellular ZIKV RNA of SH-SY5Y cells infected with either the French Polynesia or the Uganda strain and treated with 10 nM of bafilomycin A1 (pre-infection treatment). Quantification was performed by RT-qPCR after 24 hpi and the amount of ZIKV RNA was normalized to the amount of the RPL27 transcripts. Infected untreated cells were used as control; (**B**) Quantification of the amount of extracellular ZIKV RNA of SH-SY5Y cells infected with either the French Polynesia or the Uganda strain and treated with 10 nM of bafilomycin A1 (pre-infection treatment). Quantification was performed by RT-qPCR after 24 hpi and infected untreated cells served as control; (**C**) SH-SY5Y cells were infected with either the French Polynesia or the Uganda strain and treated with 10 nM of bafilomycin A1 (pre-infection treatment). Cells were fixed with 4% formaldehyde after 24 and 48 hpi and nuclei were visualized with DAPI (blue) and the ZIKV envelope protein with a specific antibody (green). Pictures were taken with the 16x objective; (**D, E**) SH-SY5Y cells were treated with 10 nM of bafilomycin A1 during 24 h (**D**) and 48 h (**E**) and the cell viability was quantified by PrestoBlue assay. Cells treated with DMSO 1:1000 and 2 % Triton X-100 (TX-100) served as vehicle and positive control, respectively. Untreated cells were used for normalization. ns = not significant $p > 0.05$; * $p \leq 0.05$; ** $p \leq 0.01$; **** $p \leq 0.0001$.

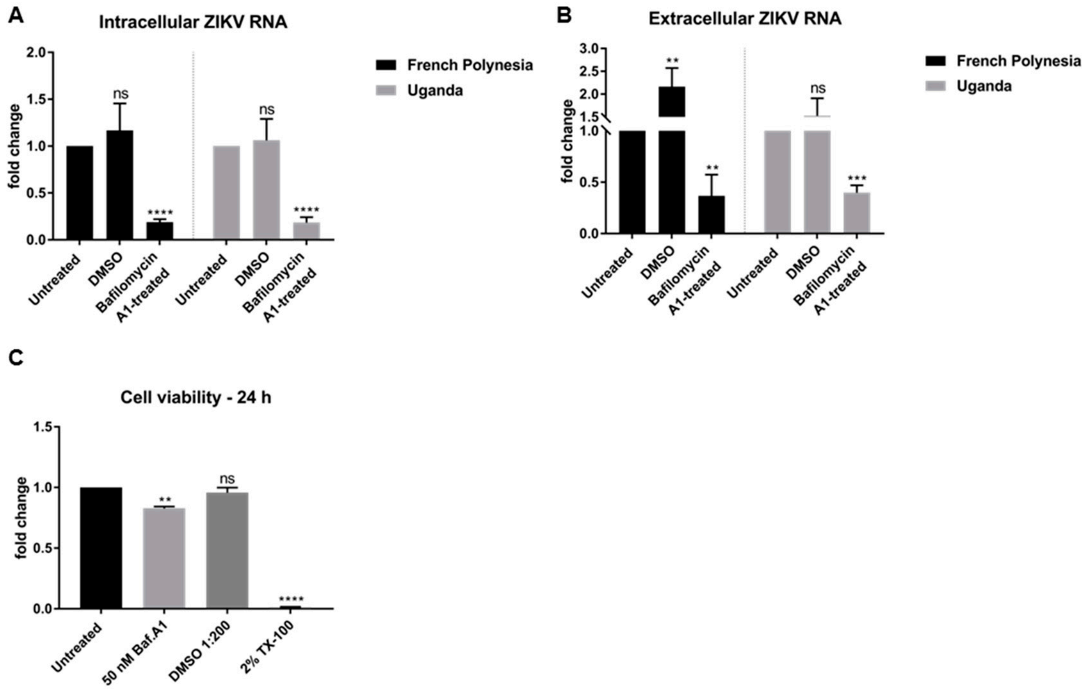

Figure S2. Bafilomycin A1 diminishes ZIKV infection in SH-SY5Y cells (**A**) Quantification of the amount of intracellular ZIKV RNA of SH-SY5Y cells infected with either the French Polynesia or the Uganda strain and treated with 50 nM of bafilomycin A1 (post-infection treatment). Quantification was performed by RT-qPCR after 72 hpi and the amount of ZIKV RNA was normalized to the amount of the RPL27 transcripts. Infected untreated cells were used as control; (**B**) Quantification of the amount of extracellular ZIKV RNA of SH-SY5Y cells infected with either the French Polynesia or the Uganda strain and treated with 50 nM of bafilomycin A1 (post-infection treatment). Quantification was performed by RT-qPCR after 72 hpi and infected untreated cells served as control; (**C**) SH-SY5Y cells were treated with 50 nM of bafilomycin A1 during 24 h and the cell viability was quantified by PrestoBlue assay. Cells treated with DMSO 1:200 and 2 % Triton X-100 (TX-100) served as vehicle and positive control, respectively. Untreated cells were used for normalization. ns = not significant $p > 0.05$; ** $p \leq 0.01$; *** $p \leq 0.001$; **** $p \leq 0.0001$.

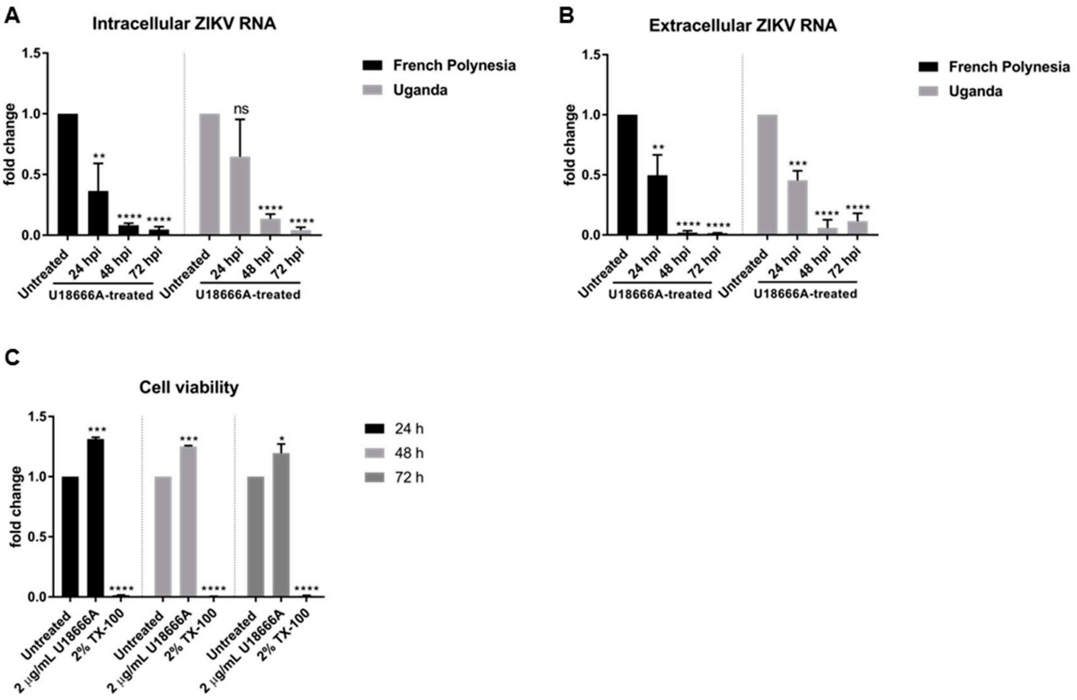

Figure S3. U18666A inhibits ZIKV infection in SH-SY5Y cells (**A**) Quantification of the amount of intracellular ZIKV RNA of SH-SY5Y cells infected with either the French Polynesia or the Uganda strain during the indicated times and treated with 2 µg/mL of U18666A (post-infection treatment). Quantification was performed by RT-qPCR and the amount of ZIKV RNA was normalized to the amount of the RPL27 transcripts. Infected untreated cells were used as control; (**B**) Quantification of the amount of extracellular ZIKV RNA of SH-SY5Y cells infected with either the French Polynesia or the Uganda strain during the indicated times and treated with 2 µg/mL of U18666A (post-infection treatment). Quantification was performed by RT-qPCR and infected untreated cells served as control; (**C**) SH-SY5Y cells were treated with 2 µg/mL of U18666A during the indicated times and the cell viability was quantified by PrestoBlue assay. Cells treated 2 % Triton X-100 (TX-100) served as positive control. Untreated cells were used for normalization. ns = not significant $p > 0.05$; * $p \leq 0.05$; ** $p \leq 0.01$; *** $p \leq 0.001$; **** $p \leq 0.0001$.

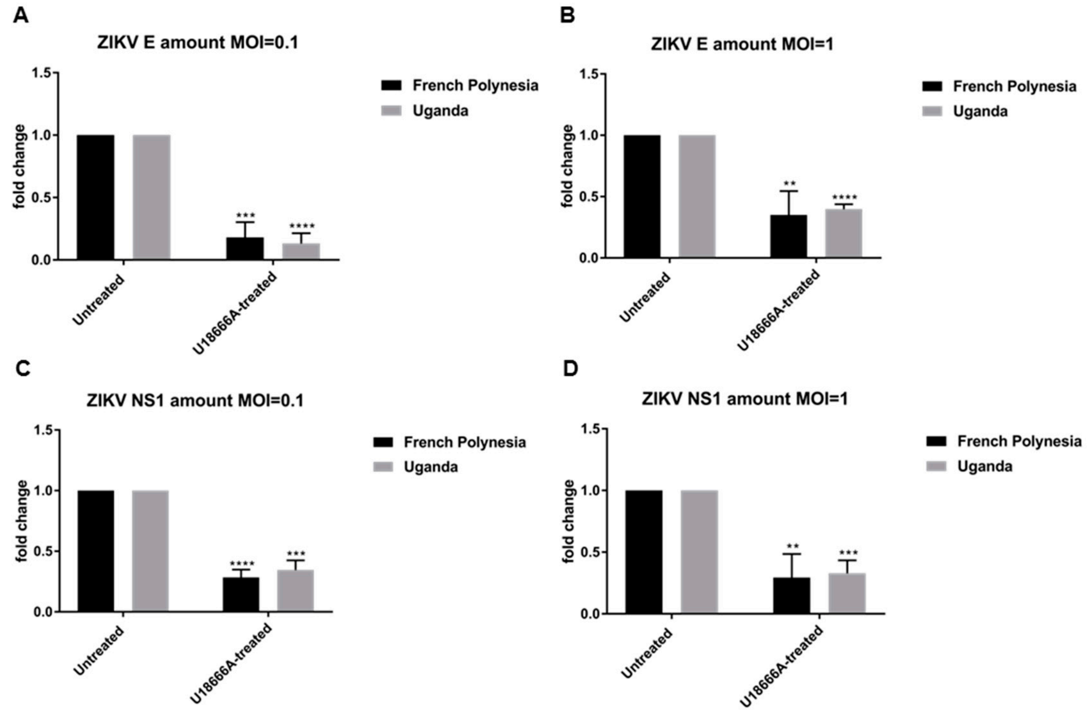

Figure S4. U18666A decreases the amount of ZIKV E and NS1 proteins in A549 cells (**A**) Densitometric quantification of the ZIKV envelope protein of SH-SY5Y cells infected with an MOI=0.1 after 16 hpi with either the French Polynesia or the Uganda strain and pre-treated with 2 μg/mL of U18666A (pre-infection treatment) for 24 h; (**B**) Densitometric quantification of the ZIKV envelope protein of SH-SY5Y cells infected with an MOI=1 after 16 hpi with either the French Polynesia or the Uganda strain and pre-treated with 2 μg/mL of U18666A (pre-infection treatment) for 24 h; (**C**) Densitometric quantification of the ZIKV NS1 protein of SH-SY5Y cells infected with an MOI=0.1 after 16 hpi with either the French Polynesia or the Uganda strain and pre-treated with 2 μg/mL of U18666A (pre-infection treatment) for 24 h; (**D**) Densitometric quantification of the ZIKV NS1 protein of SH-SY5Y cells infected with an MOI=1 after 16 hpi with either the French Polynesia or the Uganda strain and pre-treated with 2 μg/mL of U18666A (pre-infection treatment) for 24 h. Densitometric quantification of the desired proteins was accomplished by Image Studio Lite software. ** $p \leq 0.01$; *** $p \leq 0.001$; **** $p \leq 0.0001$.
